# Supplementary material for: The Effect and Cost-Effectiveness of Offering a Combined Lifestyle Intervention for the Prevention of Cardiovascular Disease in Primary Care: Results of the Healthy Heart Stepped-Wedge Trial
Source: Int J Environ Res Public Health. 2023 Mar 13;20(6):5040. doi: 10.3390/ijerph20065040 (PMC10048996; doi:10.3390/ijerph20065040)
Supplement: Supplementary file 1 [file ijerph-20-05040-s001.zip › ijerph-2203720-supplementary.pdf]

Supplementary Material File S1. Outcomes, sources and measurements of the Healthy Heart intervention.

Offering a combined lifestyle intervention for the prevention of cardiovascular disease by general practitioners: the effect and cost-effectiveness of the Healthy Heart stepped-wedge trial

Table S1. Outcomes, sources and measurements of the Healthy Heart intervention.

**Outcome**	**Measurement**	**Source**	**Frequency**
Baseline characteristics	Age, sex	Patient-reported or completed with EHR	Baseline
	Origin, educational status, living status, working status	Patient-reported	Baseline
	PCP and neighborhood	EHR	Baseline
	Neighborhood liveability index	Leefbarometer 2018(1)	Baseline
	Comorbidities†	EHR	
Determinants	Goal setting, motivation, confidence	Patient-reported	Baseline
	General Self-Efficacy scale	General Self-Efficacy Scale(2)	Baseline
	Quality of life	EQ-5D-VAS	Baseline
Primary outcomes			
Lifestyle changes	Physical activity	SQUASH (3)	Baseline/3/6/12/24 months
	Diet	DHD-FFQ, DHD-index (4)	Baseline/3/6/12/24 months
	Weight	Patient-reported, completed with EHR	Baseline/3/6/12/24 months
	Alcohol consumption	DHD-FFQ in units per day completed with average units per day registered in EHR	Baseline/3/6/12/24 months
	Smoking status	7-day + 6 months of abstinence, completed with EHR	Baseline/3/6/12/24 months

Body measurements	Weight	Patient-reported, completed with EHR	Baseline/3/6/12/24 months
	Systolic blood pressure, LDL-cholesterol, HDL-cholesterol	EHR	Baseline/3/6/12/24 months
Health care utilisation	Number of CVRM related visits to PCP in previous 6 months	Counted as a record in the EHR of an anamnestic (e.g alcohol intake), SBP measurement or laboratory test on separate dates	Baseline/6/12/24 months
Cost-effectiveness	EQ-5D-5L(5)<br>EQ-5D VAS score (6)<br>SF-12 US version(7)	Patient-reported	Baseline/6/12/24 months with 6 month recall
	Health care utilisation: number of (CVRM-related) contacts with GP, (CVRM) specialist care dietician, physiotherapist, lifestyle coach, hours of (informal) homecare, hospitalization days	Patient-reported, except for CVRM-related contacts are derived from the EHR, as described above	Baseline/6/12/24 months with 6 month recall
	CVRM telephone consultations	Laboratory measurements were counted as a telephone contact when no visit was registered during 14 days after the laboratory result, assuming the results of the measurement(s) were communicated either by telephone or	Baseline–24 months

		digital information system.	
	Medication prescription (number of days prescribed)	Derived from EHR<br>Medication included anti-coagulants (ATC codes B01AC04, clopidogrel<br>B01AC07, dipyridamole<br>B01AC24, ticagrelor<br>B01AC06, acetylsalicylic acid<br>B01AC08, carbasalate calcium<br>B01AA, vitamin K antagonists<br>B01AF, B01AE NOACs<br>diuretics (ATC C03)<br>beta blocking agents (ATC C07)<br>calcium channel blockers (C08)<br>agents acting on the renin-angiotensin system (C09) and lipid lowering medication (C10)	Baseline–24 months
	Laboratory measurements	Derived from EHR.<br>Laboratory measurements included; LDL-cholesterol, HDL-cholesterol, triglycerides, eGFR, creatinine, venous glucose, nt-pro-BNP , venous albumin concentration, albumin/creatinine ratio (urine).	Baseline–24 months

BMI, body mass index; CVRM, cardiovascular risk management, DHD-FFQ, Dutch Healthy Diet Food Frequency Questionnaire; DHD index, Dutch Healthy Diet index; EHR; routine electronic health record; EQ-5D-5L, 5-level EQ-5D; GP, general practitioner; PCP: primary care practice; QALYs, quality-adjusted life years; SF-12, short form-12; SQUASH, Short Questionnaire to Assess Health-enhancing physical activity

† comorbidities were: hypercholesterolaemia (T93), hypertension (K85, K86, K87); vascular: nephropathy (U99.01), kidney failure according to laboratory measurements (mild-high increased risk on CVD risk/progression/mortality(8)), retinopathy (F83), neuropathy (N94.02), angina pectoris (K74), myocardial infarction (K75), other ischemic diseases (K76) Transient ischemic attack (TIA) (K89), cerebral infarction (K90), peripheral artery disease (K92.01), heart failure (K77); psychiatric: alcohol or drug addiction (P15, P19), dementia (P70), anxiety and psychosis (P72, P74, P79) mood disorders (P03, P73,P76), stress (P01, P02), other (P77, P78, P80, P99, P06, P85), social problems (all ICPC codes within ‘ Z’ group) and other chronic comorbidities, based on its high prevalence in the Dutch population(9).

Measurement scales of outcomes

Origin: Dutch / Non-Dutch based on country of origin and country of origin of parents.
Educational status: low: no education/ vocational training; high: college or university
Neighborhood liveability index scoring: 1 (very weak) – 9 (excellent). Liveability index was categorised into weak (scores 1-4), satisfactory (5-6) and good–very good (7-9).
Motivation for set goal at baseline: score 0 (low motivation) – 10 (high motivation)
Confidence for set goal at baseline: score 0 (low confidence) – 10 (high confidence)
General Self-Efficacy scale: General Self-Efficacy was scored with a total score of 10 to 40. Higher scores indicate higher perceived general self-efficacy, lower scores indicate lower perceived general self-efficacy.
Quality of life: The EQ-VAS is a visual analogue scale with scores between 100 (best imaginable health) and 0 (worst imaginable health).
Dutch Healthy Diet Index: adherence to Dutch dietary guidelines: total score between 0 (no adherence) and 150 (complete adherence).
Cost prices: Cost prices for health care utilisation were derived from the Dutch reference prices for economic evaluations (8). Costs for professional home care were used as the cost price for out-of-pocket costs for basic home – and informal care.
Medication costs of generic drug were calculated as regular daily doses * number of days prescribed. Prices from a Dutch official pharmaceutical index were used (9) .
Laboratory costs were calculated with the prices from the Dutch Healthcare Authority (10) .

References

1. Leefbarometer 2018 [Internet]. Ministerie van Binnenlandse Zaken en Koninkrijksrelaties 2021. Available from: https://data.overheid.nl/dataset/leefbaarometer-meting-2018#documentation [downloaded June 2nd 2021].
2. Bosscher RJ, Smit JH. Confirmatory factor analysis of the General Self-Efficacy Scale. Behaviour research and therapy. 1998;36(3):339-43.
3. Wendel-Vos GCW, Schuit AJ, Saris WHM, Kromhout D. Reproducibility and relative validity of the short questionnaire to assess health-enhancing physical activity. Journal of clinical epidemiology. 2003;56(12):1163-9.
4. van Lee L, Feskens EJ, Meijboom S, Hooft van Huysduynen EJ, van't Veer P, de Vries JH, et al. Evaluation of a screener to assess diet quality in the Netherlands. The British journal of nutrition. 2016;115(3):517-26.
5. M. Versteegh M, M. Vermeulen K, M. A. A. Evers S, de Wit GA, Prenger R, A. Stolk E. Dutch Tariff for the Five-Level Version of EQ-5D. Value in Health. 2016;19(4):343-52.
6. Stiggelbout AM, Eijkemans MJ, Kiebert GM, Kievit J, Leer JW, De Haes HJ. The 'utility' of the visual analog scale in medical decision making and technology assessment. Is it an alternative to the time trade-off? Int J Technol Assess Health Care. 1996;12(2):291-8.
7. Brazier JE, Roberts J. The estimation of a preference-based measure of health from the SF-12. Med Care. 2004;42(9):851-9.
8. Nederlands Huisartsen Genootschap DGW, De Leest K, Schenk P, Scherpbier-De Haan N, Tjin-A-Ton J, Tuut M, Van Balen J. NHG-standaard Chronische Nierschade. 2018.
9. van Oostrom SH, Picavet HS, van Gelder BM, Lemmens LC, Hoeymans N, van Dijk CE, et al. Multimorbidity and comorbidity in the Dutch population - data from general practices. BMC public health. 2012;12:715.

Supplementary Material File S2. Missing data and sensitivity analyses.
Offering a combined lifestyle intervention for the prevention of cardiovascular disease by general practitioners: the effect and cost-effectiveness of the Healthy Heart stepped-wedge trial

Contents

1. **Table S1.** Descriptives and missing data of outcomes of the control, intervention period and persons with electronic health record data only by time measurement.
2. **Table S2.** Descriptives and missing data of cardiovascular and lifestyle behaviour outcome variables of participants and non-participants of the Healthy Heart program.
3. **Table S3.** Descriptives and missing data of cardiovascular and lifestyle behaviour outcome variables of participants of the Healthy Heart intervention with or without goal for lifestyle change.
4. **Figure S1.** Twenty-four-month trend in cardiovascular risk factor and lifestyle behaviour outcomes of the Healthy Heart intervention: participants with and without goalsetting at baseline.
5. **Table S4.** Sensitivity analysis: cardiovascular and behaviour outcomes of participants of the Healthy Heart program.
6. **Table S5.** Baseline characteristics of participants with electronic health record data only.
7. **Table S6.** Sensitivity analysis: cardiovascular and behaviour outcomes of participants in the intervention period versus participants with electronic health record data only.

Table S1. Descriptives and missing data of cardiovascular and lifestyle behaviour outcome variables of participants of the Healthy Heart study over all measuring points 0-24 months.

Outcome	Period		Baseline	3 months	6 months	12 months	24 months
**Weight (kg)**	Control	n (missing (%))	508<br>3 (0.6%)	453<br>58 (11%)	430<br>81 (16%)	443<br>68 (13%)	403<br>108 (21%)
		Mean±SD	81.4±15.8	81.5±14.9	81.0±14.5	81.2±14.9	80.8±14.5
	Intervention	n (missing (%))	271.0<br>5 (1.8%)	231.0<br>45 (16%)	199.0<br>77 (28%)	205.0<br>71 (26%)	193.0<br>83 (30%)
		Mean±SD	82.8±17.0	82.2±17.0	80.6±17.1	81.3±18.0	80.9±17.1
	Only EHR	n (missing (%))	142.0<br>13 (8.4%)	27.0<br>128 (83%)	43.0<br>112 (72%)	95.0<br>60 (39%)	80.0<br>75 (48%)
		Mean±SD	81.8±16.5	81.2±18.4	84.3±14.9	79.9±15.7	81.5±13.8
**SBP mmHg**	Control	n (missing (%))	417<br>94 (18%)	113<br>398 (78%)	191<br>320 (63%)	289<br>222 (43%)	228<br>283 (55%)
		Mean±SD	136±15	138±16	135±13	134±13	136±13
	Intervention	n (missing (%))	212<br>64 (23%)	41<br>235 (85%)	65<br>211(76%)	144<br>132 (48%)	99<br>177 (64%)
		Mean±SD	136±15	135±17	137±14	134±14	136±14
	Only EHR	n (missing (%))	147<br>8 (5.2%)	28<br>127 (82%)	44<br>111 (72%)	99<br>56 (36%)	80<br>75 (48%)
		Mean±SD	136±15	142±12	136±16	134±14	133±16
		n	276	84	116	241	201

**LDL-cholesterol**	Control	(missing (%))	235 (46%)	427 (84%)	395 (77%)	270 (53%)	310 (61%)
		Mean±SD	3.3±1.0	3.0±0.8	3.1±0.9	3.1±0.9	3.1±1.0
	Intervention	n<br>(missing (%))	171<br>105 (38%)	30<br>246 (89%)	48<br>228 (83%)	141<br>135 (49%)	99<br>177 (64%)
		Mean±SD	3.2±0.9	3.3±1.1	3.2±1.1	3.1±0.9	3.0±0.8
	Only EHR	n<br>(missing (%))	117<br>38 (25%)	16<br>139 (90%)	32<br>123 (79%)	88<br>67 (43%)	71<br>84 (54%)
		Mean±SD	3.2±0.8	3.6±0.9	3.0±0.9	3.0±0.9	3.0±0.8
**HDL-cholesterol**	Control	n<br>(missing (%))	275.0<br>236 (46%)	84.0<br>427 (84%)	114.0<br>397 (78%)	242.0<br>269 (53%)	200.0<br>311(61%)
		Mean±SD	1.5±0.4	1.4±0.5	1.5±0.4	1.5±0.5	1.5±0.5
	Intervention	n<br>(missing (%))	170.0<br>106 (38%)	30.0<br>246 (89%)	47.0<br>229 (83%)	142.0<br>134 (49%)	101.0<br>175 (63%)
		Mean±SD	1.5±0.4	1.4±0.4	1.4±0.3	1.5±0.4	1.5±0.4
	Only EHR	n<br>(missing (%))	117.0<br>38 (25%)	16.0<br>139 (90%)	32.0<br>123 (79%)	90.0<br>65 (42%)	74.0<br>81 (52%)
		Mean±SD	1.5±0.5	1.4±0.5	1.5±0.5	1.5±0.4	1.5±0.4
**Minutes of weekly physic**	Control	n<br>(missing (%))	477<br>34 (6.7%)	429<br>82 (16%)	392<br>119 (23%)	362<br>149 (29%)	317<br>194 (38%)
		Mean±SD	1997±1138	1924±1178	1944±1272	1922±1157	1770±1124

**al activity †**	Intervention	n<br>(missing (%))	253<br>14 (8.3%)	220<br>56 (20%)	171<br>105<br>(38%)	156<br>120<br>(44%)	155<br>121<br>(44%)
		Mean±SD	2085±1279	1964±1298	1982±1218	1985±1283	1942±1238
**DHD index †**	Control	n<br>(missing (%))	450<br>61 (12%)	396<br>115<br>(23%)	346<br>165<br>(32%)	335<br>176<br>(34%)	293<br>218<br>(43%)
		Mean±SD	99±18	104±18	104±18	106±18	105±18
	Intervention	n<br>(missing (%))	246<br>30 (11%)	207<br>69 (25%)	161<br>115<br>(42%)	148<br>128<br>(46%)	145<br>131<br>(47%)
		Mean±SD	100±20	105±20	106±21	107±19	108±18
**Alcohol intake (average number of units per day)**	Control	n<br>(missing (%))	500<br>11 (2.2%)	445<br>66 (13%)	420<br>91 (18%)	431<br>80 (16%)	390<br>121(24%)
		Median (IQR)	0.7 (0.1-1.7)	0.7 (0.1-1.7)	0.6 (0.0-1.4)	0.7 (0.0-1.7)	0.6 (0.0-1.7)
	Intervention	n<br>(missing (%))	269<br>7 (2.5%)	233<br>43 (16%)	192<br>84 (30%)	195<br>81 (29%)	187<br>89 (32%)
		Median (IQR)	0.6 (0.0-1.4)	0.6 (0.0-1.1)	0.7 (0.0-1.4)	0.6 (0.0-1.1)	0.6 (0.0-1.4)
	EHR only	n<br>(missing (%))	103<br>52 (34%)	12<br>143<br>(92%)	30<br>125<br>(81%)	79<br>76 (49%)	65<br>90 (58%)
		Median (IQR)	1.0 (0.0-2.0)	0.0 (0.0-0.8)	1.0 (0.0-1.3)	1.0 (0.0-2.0)	0.0 (0.0-1.0)
**Smoking * (quit smoking=yes)**	Control	n<br>(missing (%))	0 (0)	6<br>9 (21%)	5<br>12 (28%)	0<br>15 (35%)	7<br>20 (47%)
	Intervention	n<br>(missing (%))	0 (0)	3<br>4 (21%)	2<br>9 (47%)	3<br>9 (47%)	1<br>11 (58%)

**Number of CVRM related contacts with PCP†**	Control	n (missing (%))	60 (12%)	60 (12%)	60 (12%)	60 (12%)	60 (12%)
		Median (IQR)	1 (1-2)	1 (0-2)	1 (1-2)	1 (0-2)	1 (0-2)
	Intervention	n (missing (%))	32 (12%)	32 (12%)	32 (12%)	32 (12%)	32 (12%)
		Median (IQR)	2 (1-2)	1 (0-2)	1 (0-2)	1 (0-2)	1 (0-2)
	EHR only	n (missing (%))	155<br>0	155<br>0	155<br>0	155<br>0	155<br>0
		Median IQR)	1 (0-2)	1(1-2)	1 (0.75-2)	1 (0-1)	1 (0-2)

† not available for EHR group

* numbers of participants that quit smoking during 7 days (3 and 6 months) and 6 months (12 and 24 months). Number of participants that indicated current smoking at baseline control period= 43 (69%), intervention period=19 (31%) of total n=62.

Table S2. Descriptives and missing data of cardiovascular and lifestyle behaviour outcome variables of participants and non-participants of the Healthy Heart program over all measuring points 0-24 months.

Outcome	Period		Baseline	3 months	6 months	12 months	24 months
**Weight (kg)**	Non-participants	n (missing (%))	740 (0.9%)	652 (13%)	602 (19%)	620 (17%)	569 (24%)
		Mean±SD	81.2±15.6	81.1±15.0	80.4±14.6	80.8±15.5	80.3±14.6
	Participants	n (missing (%))	1 (2.5%)	8 (20%)	13 (33%)	12 (30%)	13 (33%)
		Mean±SD	94.5±22.1	94.1±23.0	92.7±25.2	90.2±23.4	92.2±24.6
**SBP mmHg**	Non-participants	n (missing (%))	594 (20%)	142 (81%)	243 (67%)	409 (45%)	311 (58%)
		Mean±SD	136±15	137±16	135±13	134±14	136±14
	Participants	n (missing (%))	35 (13%)	12 (70%)	13 (68%)	24 (40%)	16 (60%)
		Mean±SD	140±16	137±15	136±14	137±18	141±17
**Minutes of weekly physical activity**	Non-participants	n (missing (%))	693 (7.2%)	618 (17%)	541 (28%)	500 (33%)	451 (40%)
		Mean±SD	2064±1229	1957±1255	1982±1236	1976±1245	1885±1197

	Participants	n (missing (%))	37 (7.5%)	31 (23%)	22 (45%)	18 (55%)	21 (48%)
		Mean±SD	1875±1296	1827±1341	1695±1180	1696±1288	1907±1364
**LDL-cholesterol**	Non-participants	n (missing (%))	422 (44%)	107 (86%)	148 (80%)	360 (52%)	288 (61%)
		Mean±SD	3.3±1.0	3.0±0.8	3.1±0.9	3.1±0.9	3.1±0.9
	Participants	n (missing (%))	25 (38%)	7 (83%)	16 (60%)	22 (45%)	12 (70%)
		Mean±SD	3.2±0.68	3.9±1.5	3.3±1.2	3.2±1.0	2.9±1.1
**HDL-cholesterol**	Non-participants	n (missing (%))	420 (44%)	107 (86%)	146 (80%)	362 (52%)	289 (61%)
		Mean±SD	1.5±0.4	1.4±0.4	1.5±0.4	1.5±0.4	1.5±0.4
	Participants	n (missing (%))	25 (38%)	7 (83%)	15 (63%)	22 (45%)	12 (70%)
		Mean±SD	1.5±0.4	1.4±0.4	1.4±0.3	1.5±0.4	1.5±0.2
**DHD index**	Non-participants	n (missing (%))	660 (12%)	573 (23%)	485 (35%)	466 (38%)	416 (44%)
		Mean±SD	99±18	104±19	104±19	106±18	106±19
	Participants	n (missing (%))	36 (10%)	30 (25%)	22 (45%)	17 (58%)	22 (45%)

		Mean±SD	96±16	111±17	111±14	109±18	111±15
**Alcohol intake (average number of units per day)**	Non-participants	n (missing (%))	730 (2.3%)	644 (14%)	586 (22%)	601 (20%)	552 (26%)
		Median (IQR)	0.7 (0.1-1.6)	0.7 (0.1-1.5)	0.7 (0.0-1.4)	0.6 (0.0-1.4)	0.6 (0.0-1.4)
	Participants	n (missing (%))	39 (2.5%)	34 (15%)	26 (35%)	25 (38%)	25 (38%)
		Median (IQR)	0.4 (0.0-0.9)	0.3 (0.0-0.9)	0.2 (0.0-1.0)	0.3 (0.0-1.0)	0.4 (0.0-1.7)
**Quit smoking = yes***	Participants	n (missing (%))	0 (0)	2<br>1 (33%)	1<br>2 (67%)	1<br>2 (67%)	1<br>2 (67%)
	Non-participants	n (missing (%))	0 (0)	7<br>12 (20%)	6<br>19 (32%)	2<br>22 (37%)	8<br>29 (49%)
**Number of CVRM related contacts with PCP†**	Participants	n (missing (%))	608 (19%)	473 (37%)	511(32%)	464 (38%)	455 (39%)
		Median (IQR)	1 (1-2)	1 (1-2)	1 (1-2)	1 (1-2)	1 (1-2)
	Non-participants	n (missing (%))	37(7.5%)	31(23%)	27(33%)	27(33%)	27(33%)

		Median (IQR)	1 (2-2)	2 (1-3)	2 (1-3)	1 (1-3)	2 (1-2)
* number of current smokers at baseline (n=62), participants HH program n=3, non-participants n=59							

Table S3. Descriptives and missing data of cardiovascular and lifestyle behaviour outcome variables of participants of the Healthy Heart intervention with or without goal for lifestyle change over all measuring points 0-24 months.

Outcome	Group		Baseline	3 months	6 months	12 months	24 months
**Weight (kg)**	With goal	n (missing (%))	455 (0%)	398 (13%)	358 (21%)	367 (19%)	330 (27%)
		Mean±SD	82.9±16.1	82.4±15.4	83.1±15.3	85.9±15.1	82.0±15.3
	Without goal	n (missing (%))	286 (0%)	248 (13%)	238 (17%)	253 (12%)	237 (17%)
		Mean±SD	73.5±12.4	73.7±12.3	73.7±12.2	73.8±14.1	73.5±12.3
**Minutes of weekly physical activity**	With goal	n (missing (%))	459 (2.1%)	382 (7.2%)	328 (25%)	293 (33%)	272 (37%)
		Mean±SD	2089±1270	1980±1277	2041±1275	2029±1196	1923±1238
	Without goal	n (missing (%))	268 (1.5%)	234 (2.6%)	211 (19%)	205 (21%)	183 (28%)
		Mean±SD	1997±1168	1878±1878	1863±1863	1867±1867	1774±1774
**DHD index**	With goal	n (missing %))	427 (6.4%)	350 (12%)	297 (31%)	271 (36%)	250 (40%)
		Mean±SD	99±18	106±19	106±20	107±18	106±19

	Without goal	n (missing (%))	267 (6.0%)	228 (8.5%)	193 (27%)	194 (27%)	174 (34%)
		Mean±SD	100±18	103±19	104±18	104±18	105±18
**Alcohol intake (average number of units per day)**	With goal	n (missing (%))	160 (0%)	134 (16%)	128 (20%)	127 (21%)	118 (26%)
		Median (IQR)	1.4 (0.9-2.6)	1.3 (0.6-2.4)	1.3 (0.7-2.1)	1.1 (0.6-2.0)	1.0 (0.3-2.0)
	Without goal	n (missing (%))	416 (0%)	367 (12%)	327 (21%)	346 (17%)	311 (26%)
		Median (IQR)	0.9 (0.3-1.6)	0.9 (0.3-1.4)	0.9 (0.3-1.4)	0.9 (0.3-1.4)	0.9 (0.3-1.4)
**Quit smoking = yes**	With goal	n (n missing (%))	30 (0)	6 (5 (16%)	2 (8 (27%)	1 (11 (37%)	3 (15 (50%)
	Without goal	n (n missing (%))	32 (0)	3 (8 (25%)	5 (5 (16%)	2 (13 (41%)	5 (16 (50%)

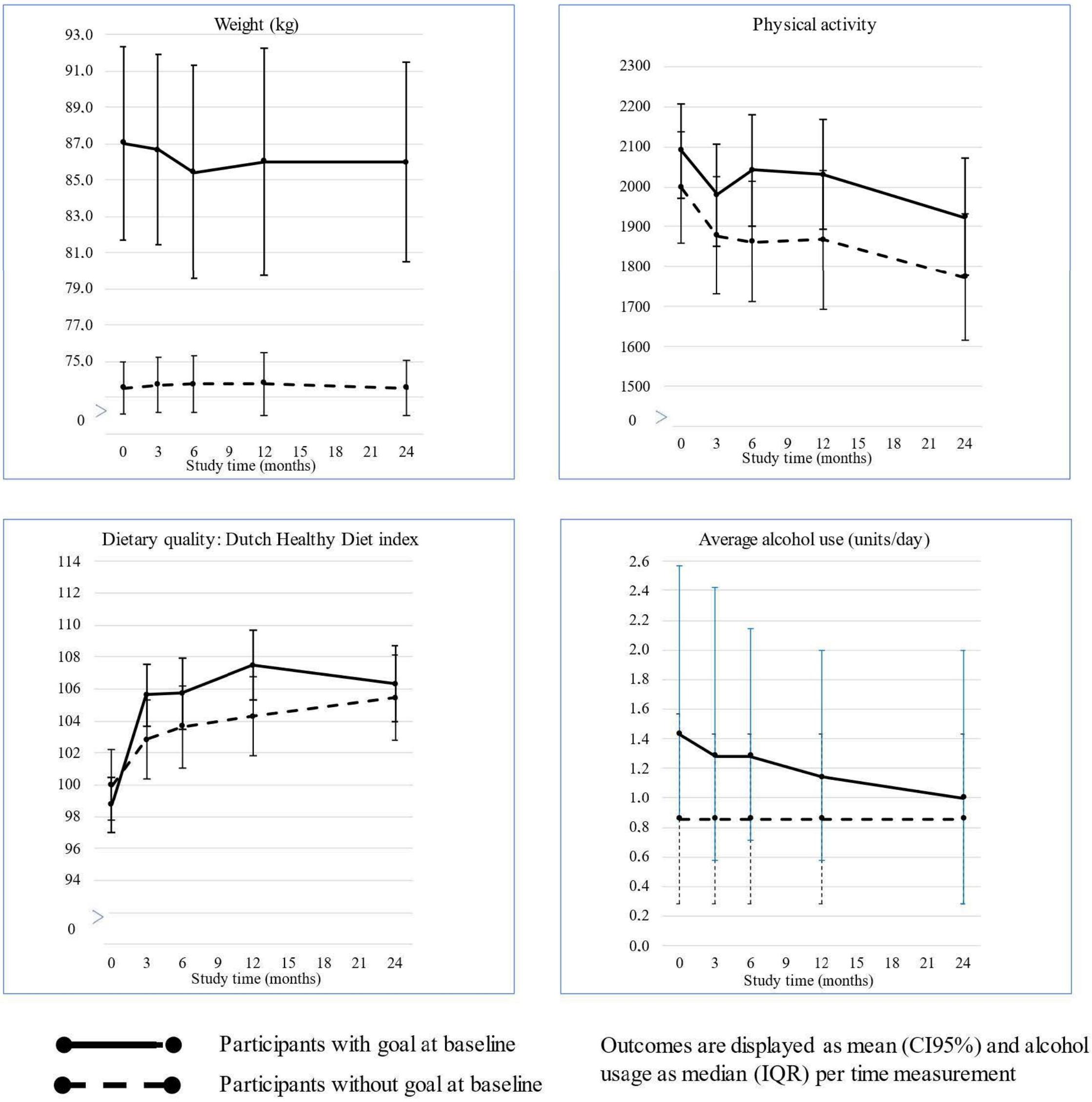

Figure S1. Twenty-four-month trend in cardiovascular risk factor and lifestyle behaviour outcomes of the Healthy Heart intervention: participants with and without goalsetting at baseline.

Table S4. Cardiovascular and lifestyle behaviour outcomes of participants of the Healthy Heart study: difference between participants and non-participants of Healthy Heart program.

Outcome	Baseline mean±SD, median (IQR) or n (%) [a]		Between-group difference: β or odds (CI95%)[b]	
	Non-participants (n=747)	Participants (n=40)	3-6 months	12-24 months
**Weight** (kg)	81.2±15.6	94.5±22.1	0.29 (-0.88-1.46)	-0.41 (-2.23-1.41)
**Systolic blood pressure** (mmHg)	136±15	140±16	-2.68 (-7.61-2.25)	1.23 (-2.60-5.08)
**LDL-cholesterol (mmol/l)**	3.26<br>0.97	3.21±0.68	0.14 (-0.35-0.62)	0.12 (-0.15-0.39)
**HDL-cholesterol**	1.49±0.43	1.48±0.44	-0.02 (-0.15-0.10)	**-0.09 (-0.17—0.01)**
**Minutes of weekly physical activity**	2064±<br>1228	1875±<br>1296	-43 (-348-263)	84 (-308-475)
**DHD index**	99.5±18.3	95.8±16.3	**8.75 (4.64-12.86)**	**7.29 (2.34-12.23)**
**Alcohol intake** [c]<br>No alcohol<br>0-1<br>1-2<br>2 or more	 175 (24%)<br>291 (39%)<br>149 (20%)<br>115 (16%)	 10 (25%)<br>22 (55%)<br>3 (7.5%)<br>4 (10%)	**0.66 (0.65-0.66)**	0.89 (0.08-9.66)
**Quit smoking** [d]	59 (7.9%)	3 (7.5%)	n/a	n/a
**Number of CVRM related contacts with PCP in the past 6 months**	1 (1-2)	2 (1-2)	**0.38 (0.20-0.55)**	**1.52 (1.12-2.05)**
**QALY**				

[a] percentages are of all participants per period
[b] reference group: non-participants
Models are adjusted for baseline value of outcome
For alcohol intake and number of CVRM visits, between-group differences are displayed in odds ratio (CI95%)
[c] Alcohol: average units per day; percentages are of all participants per period; reference group: no alcohol; models are not adjusted for PCP, due to lack of power
[d] Smoking: numbers of current smoking participants and non-participants of the Healthy Heart program. Due to the small numbers of smoking participants in the program, odds ratios could not be estimated. Number of participants who quit smoking were n (% of total): participants: 2 (66%) (3-6 months) and 0 (0%) (12-24 months) and non-participants: 4 (6.8%) (3-6 months) and 4 (6.8%) (12-24 months).
Bold indicates a significant between-group difference p<0.05
DHD-index: Dutch Healthy Diet index, PCP: primary care practice

Table S5. Baseline characteristics of included study participants with electronic health record data only.

Characteristic	Only EHR data (N=155)	Available data (n)
**Age** (years)	66.4±9.1	149
**Sex** (women)	71 (47%)	150
**Origin** (Dutch)	*n/a*	*n/a*
**Educational level** (low)	*n/a*	*n/a*
**Household composition** (cohabiting)	*n/a*	*n/a*
**Job status**<br>Currently employed (< 65 years)<br>not employed (<65 years)<br>retirement (> 65 years)	*n/a*	*n/a*
**Neighbourhood deprivation score**<br>Weak<br>Satisfactory<br>Good–Extremely good	 24 (16%)<br>23 (15%)<br>108 (69%)	155
**Comorbidity***<br>Chronic comorbidity<br>Hypercholesterolaemia<br>Hypertension<br>Macrovascular disease<br>Impaired kidney function	 17 (11%)<br>55 (36%)<br>132 (85%)<br>6 (3.9%)<br>30 (19%)	155
**Medication usage (yes)**<br>Antihypertensive<br>Lipid lowering<br>Anti coagulants or thrombocyte aggregation inhibitors<br>Psychiatric medication	89 (57%)<br>54 (35%)<br>11 (7.1%)<br>23 (15%)	155
**Body mass index (kg/m²)**	27.5±5.0	141
**Use of alcohol** (yes)	83 (66%)	125
**Smoking status**<br>Never<br>Previous<br>Current	 50 (50%)<br>34 (34%)<br>15 (15%)	125
**General self-efficacy scale (scale 10-40)**	*n/a*	*n/a*
**Quality of life (scale 0-100)**<br>Perceived health, EQ-VAS score	*n/a*	*n/a*
**Adherence to exercise guideline (yes)**<br>minutes of physical activity<br>minutes of physical activity + strength and balance	*n/a*	*n/a*

Continuous values are presented as means ± standard deviation or median (IQR) (interquartile range)

Categorical values are presented as n (% of participants with non-missing data within group)

** Comorbidities were: hypercholesterolaemia (T93), hypertension (K85, K86, K87); vascular: nephropathy (U99.01), kidney failure according to laboratory measurements (mild-high increased risk on CVD risk/progression/mortality(9)), retinopathy (F83), neuropathy (N94.02), angina pectoris (K74), myocardial infarction (K75), other ischemic diseases (K76) Transient ischemic*

attack (TIA) (K89), cerebral infarction (K90), peripheral artery disease (K92.01), heart failure (K77); psychiatric: alcohol or drug addiction (P15, P19), dementia (P70), anxiety and psychosis (P72, P74, P79) mood disorders (P03, P73,P76), stress (P01, P02), other (P77, P78, P80, P99, P06, P85), social problems (all ICPC codes within ' Z' group) and other chronic comorbidities, based on its high prevalence in the Dutch population(10).

VAS: Visual Analogue Scale

Table S6. Cardiovascular and lifestyle behaviour outcomes of participants of the Healthy Heart study: difference between participants in intervention period and participants with only health record data (EHR-group).

**Outcome**	**Baseline mean±SD, median (IQR) or n (%)**[a]		**Between-group difference: β or odds (CI95%)**[b]
	**Participants with EHR data only (n=155)**	**Intervention period (n=276)**	**12-24 months**
**Weight** (kg)	81.8±16.5	82.8±17.0	1.01 (-0.73-2.76)
**Systolic blood pressure** (mmHg)	136±15	136±15	2.05 (-0.51-4.61)
**LDL-cholesterol (mmol/l)**	3.2±0.8	3.2±0.9	-0.002 (-0.17-0.17)
**HDL-cholesterol**	1.5±0.5	1.5±0.4	-0.001 (-0.05-0.05)
**Number of CVRM related contacts with PCP in the past 6 months**	1 (0-2)	2 (1-2)	0.94 (0.73-1.20)

[a] percentages are of all participants per period
[b] reference group: Participants with EHR data only
Models 3-6 months are adjusted for baseline value of outcome, models 12-24 months are additionally adjusted for age and sex (weight, blood pressure, HDL-cholesterol, alcohol) or lipid lowering medication (LDL-cholesterol)
For alcohol intake and number of CVRM visits, between-group differences are displayed in odds ratio (CI95%)
Bold indicates a significant between-group difference $p<0.05$
EHR: electronic health record, CVRM: cardiovascular risk management, PCP: primary care practice

Supplementary Material File S3. Economic evaluation.

Offering a combined lifestyle intervention for the prevention of cardiovascular disease by general practitioners: the effect and cost-effectiveness of the Healthy Heart stepped-wedge trial

Contents

Table S1. Missing data.
Results of the economic evaluation.
Table S2. QALYs.
Table S3. Costs control versus intervention period.
Table S4. Costs Healthy Heart program.
Figure S1. Cost-effectiveness acceptability curves.

Table S1. Missing data of the cost-effectiveness variables per period of the Healthy Heart trial.

	6 months prior to baseline		0-6 months after baseline		6-12 months after baseline		12-18 months after baseline		18-24 months after baseline	
	N	Missing (%)	N	Missing (%)	N	Missing (%)	N	Missing (%)	N	Missing (%)
SF12 1 General health	731	55 (7.0%)	565	221 (28.1%)	521	265 (33.7%)	0	786 (100.0%)	471	315 (40.1%)
SF12 2 Activities / limitations	728	58 (7.4%)	562	224 (28.5%)	519	267 (34.0%)	0	786 (100.0%)	467	319 (40.6%)
SF12 3 Activities / physical health	723	63 (8.0%)	556	230 (28.3%)	514	272 (34.6%)	0	786 (100.0%)	466	320 (40.7%)
SF12 4 physical: less accomplished	725	61 (7.8%)	559	227 (28.9%)	513	273 (34.7%)	0	786 (100.0%)	452	334 (42.5%)
SF12 4b Physical: Limited in kind of activities	720	66 (8.4%)	553	233 (28.6%)	511	275 (35.0%)	0	786 (100.0%)	464	322 (41.0%)
SF12 5a Mental: accomplished less	730	56 (7.1%)	563	223 (28.4%)	513	273 (34.7%)	0	786 (100.0%)	462	324 (41.2%)
SF12 5b Mental: less carefully	725	61 (7.8%)	554	232 (28.5%)	507	279 (35.5%)	0	786 (100.0%)	455	331 (42.1%)
SF12 6 Pain	731	55 (7.0%)	560	226 (28.8%)	520	266 (33.8%)	0	786 (100.0%)	471	315 (40.1%)
SF12 7a Calm	730	56 (7.1%)	562	224 (28.5%)	520	266 (33.8%)	0	786 (100.0%)	470	316 (40.2%)
SF12 7b Energy	730	56 (7.1%)	562	224 (28.5%)	520	266 (33.8%)	0	786 (100.0%)	471	315 (40.1%)
SF12 7c Down-hearted	729	57 (7.3%)	562	224 (28.5%)	519	267 (34.0%)	0	786 (100.0%)	469	317 (40.3%)
SF12 8 Social activities	732	54 (6.9%)	563	223 (28.4%)	520	266 (33.8%)	0	786 (100.0%)	471	315 (40.1%)
EQ-5D Mobility	731	55 (7.0%)	564	222 (28.2%)	522	264 (33.6%)	0	786 (100.0%)	474	312 (38.7%)
EQ-5D Selfcare	727	59 (7.5%)	562	224 (28.5%)	518	268 (34.1%)	0	786 (100.0%)	472	314 (38.9%)
EQ-5D	730	56 (7.1%)	564	222 (28.2%)	521	265 (33.7%)	0	786 (100.0%)	474	312 (38.7%)

Daily activities										
EQ-5D Pain	731	55 (7.0%)	563	223 (28.4%)	522	264 (33.6%)	0	786 (100.0%)	473	313 (38.8%)
EQ-5D Mood	730	56 (7.1%)	564	222 (28.2%)	521	265 (33.7%)	0	786 (100.0%)	472	314 (38.9%)
EQ-5D VASSCORE	730	56 (7.1%)	563	223 (28.4%)	521	265 (33.7%)	0	786 (100.0%)	466	320 (40.7%)
Selfreported Contacts PCP	730	56 (7.1%)	562	224 (28.5%)	519	267 (34.0%)	0	786 (100.0%)	471	315 (40.1%)
EHR: Telephone consultations	683	103 (13.1%)	683	103 (13.1%)	683	103 (13.1%)	683	103 (13.1%)	683	103 (13.1%)
EHR: GP consultations	691	95 (12.1%)	691	95 (12.1%)	691	95 (12.1%)	691	95 (12.1%)	691	95 (12.1%)
EHR: PN consultations	691	95 (12.1%)	689	97 (12.3%)	689	97 (12.3%)	683	103 (13.1%)	688	98 (12.5%)
Lifestyle coach	731	55 (7.0%)	562	224 (28.5%)	515	271 (34.5%)	0	786 (100.0%)	468	318 (40.5%)
Other coach	731	55 (7.0%)	562	224 (28.5%)	515	271 (34.5%)	0	786 (100.0%)	469	317 (40.3%)
Dietician	731	55 (7.0%)	565	221 (28.1%)	520	266 (33.8%)	0	786 (100.0%)	471	315 (40.1%)
Physiotherapist	730	56 (7.1%)	563	223 (28.4%)	519	267 (34.0%)	0	786 (100.0%)	471	315 (40.1%)
Specialist consultation	730	56 (7.1%)	563	223 (28.4%)	520	266 (33.8%)	0	786 (100.0%)	472	314 (38.9%)
Hospital stay	731	55 (7.0%)	565	221 (28.1%)	518	268 (34.1%)	0	786 (100.0%)	471	315 (40.1%)
Home care	731	55 (7.0%)	565	221 (28.1%)	517	269 (34.2%)	0	786 (100.0%)	471	315 (40.1%)
Informal care	731	55 (7.0%)	565	221 (28.1%)	517	269 (34.2%)	0	786 (100.0%)	471	315 (40.1%)
Laboratory costs	691	95 (12.1%)	689	97 (12.3%)	689	97 (12.3%)	683	103 (13.1%)	688	98 (12.5%)
Medication costs	691	95 (12.1%)	689	97 (12.3%)	689	97 (12.3%)	683	103 (13.1%)	688	98 (12.5%)
Age	783	3 (0.4%)	783	3 (0.4%)	783	3 (0.4%)	783	3 (0.4%)	783	3 (0.4%)
Sex	784	2 (0.3%)	784	2 (0.3%)	784	2 (0.3%)	784	2 (0.3%)	784	2 (0.3%)
Postal code	741	45 (5.7%)	741	45 (5.7%)	741	45 (5.7%)	741	45 5.7%)	741	45 (5.7%)
Baseline systolic blood pressure (mmHg%)	629	157 (20.0%)	629	157 (20.0%)	629	157 (20.0%)	629	157 (20.0%)	629	157 (20.0%)

Baseline LDL-cholesterol (mmol/mol %)	447	339 (43.1%)	447	339 (43.1%)	447	339 (43.1%)	447	339 (43.1%)	447	339 (43.1%)
Baseline body mass index (kg/m2%)	778	8 (1.0%)	778	8 (1.0%)	778	8 (1.0%)	778	8 (1.0%)	778	8 (1.0%)

PCP: primary care practice, GP: general practitioner, PN: practice nurse, VAS: visual analogue scale
SF-6D: Short-Form-12 questionnaire item
EQ-5D: EuroQol 5D questionnaire item

Missing data imputation procedure

For the cost-effectiveness analysis missing data was imputed using multiple imputation(1) using the procedure of SPSS version 25.0, with the fully conditional specification setting using predictive mean matching for continuous data, and binary logistic and poly-logistic modelling for categorical data, with 10 iterations and the creation of 100 imputed datasets. Predictors in the imputation procedure included inclusion period (control/intervention), sex, age, utilities, medication costs, GP cardiovascular risk management (CVRM) costs, GP other costs and total medical specialist outpatient costs. For rarely used care (n=<30; GP home visits, non-CVRM related hospitalisation, hospital number), predictors were inclusion period, sex, age and utilities; for very rarely used care (n<15; CVRM related hospitalisation, hospital type, lifestyle coaching, home care), only inclusion period. Informal home care at 24 months was imputed based on only baseline informal home care as it was extremely rare (<5).

1. Faria R, Gomes M, Epstein D, White IR. A Guide to Handling Missing Data in Cost-Effectiveness Analysis Conducted Within Randomised Controlled Trials. PharmacoEconomics. 2014;32(12):1157-70.

Results of the economic evaluation

Participants' utilities at baseline were comparable between the control and intervention period (adjusted mean difference (95% CI) -0.004 (-0.04;0.03)) (Table 1, main paper). First-year QALYs were significantly lower in the participants in the intervention group (mean difference (95% CI) -0.06 (-0.11;-0.02)), while in the second year the difference was non-significant (Supplementary Material (SM) Table S2,). Also for the total study period, QALYs were lower in participants during the intervention period, but the association did not reach significance (intervention: 1.38±0.88, control: 1.49±0.79, adjusted difference (95%CI) -0.10 (-0.20;0.002)). QALYs based on the SF-6D questionnaire and EQ-VAS showed similar results (Figure 3 and SM C).

Costs

Baseline costs were comparable between the periods (adjusted mean difference (CI95%) -18 (-247;212) p=0.881) (Table 1, main paper). With 15% of the patients participating in the Healthy Heart program, average intervention costs were estimated at EUR 91 per patient in the intervention period (CI95% 70-108) (SM C, Table S4). Mean costs of 2-year total CVRM care were comparable (mean±SD intervention: mean adjusted difference (CI95%) EUR 106 (-80; 293) p=0.266).Mean 2-year total health care costs were mean±SD EUR 2909±4826 (intervention) and EUR 2457±3494 (control), with no significant difference between the periods (mean adjusted difference (CI95%) EUR 484 (-110;1079) p=0.110) (SM C, Table S3).

Combining the difference in QALYs and costs, the probability that offering the Healthy Heart program in general practices is cost-effective compared to not offering the program was below 6%, regardless of the willingness-to-pay per QALY (SM C, Figure S1A). In the sensitivity analyses using the SF-6D and EQ-VAS to calculate QALYs, this probability remained below 7% (SF-6D) and 26% (EQ-VAS). In the sensitivity analysis using the willingness-to-pay for cardiovascular risk management care costs, this probability remained below 14% (EQ-5D and SF-12) and 35% (EQ-VAS) (SM C, Figure S1B).

Table S2. QALYs for the intervention and the control period.

	Period		Mean adjusted difference (95% CI)	p-value
**QALY**	Intervention (n=275)	Control (n=511)	Reference: control period	
**Baseline utility**				
EQ-5D-5L	0.85±0.28	0.85±0.32	-0.004 (-0.04;0.03)	0.836
SF-6D	0.80±0.13	0.81±0.14	-0.01 (-0.03;0.01)	0.408
VAS	0.90±0.12	0.91±0.11	-0.01 (-0.03;0.01)	0.171
**0-12 months QALYs**				
EQ-5D-5L	0.70±0.46	0.77±0.38	**-0.06 (-0.11;-0.02)**	0.006
SF-6D	0.76±0.19	0.79±0.17	-0.02 (-0.04;-0.001)	0.067
VAS	0.91±0.10	0.92±0.09	-0.002 (-0.01;0.002)	0.724
**12-24 months QALYs**				
EQ-5D-5L	0.68±0.59	0.72±0.56	-0.04 (-0.11;0.04)	0.364
SF-6D	0.74±0.29	0.76±0.33	-0.01 (-0.04;0.01)	0.314
VAS	0.91±0.10	0.92±0.10	-0.001 (-0.02;0.01)	0.837
**0-24 months QALYs**				
EQ-5D-5L	1.38±0.88	1.49±0.79	-0.10 (-0.20;0.002)	0.55
SF-6D	1.50±0.41	1.55±0.43	-0.03 (-0.07;0.008)	0.116
VAS	1.82±0.19	1.84±0.17	-0.004 (-0.03;0.02)	0.753

Comparisons are adjusted for sex, age, baseline utility and baseline total healthcare costs
Outcomes are pooled results of 100 imputed datasets
Bold indicates p-value <0.05

Table S3. Mean health care utilisation and costs Healthy Heart trial (24-months follow-up).

	**Intervention period (n=275)**		**Control period (n=511)**		**Difference**	
	Volumes (visits)	Costs	Volumes (visits)	Costs	Adjusted difference in costs*	p-value
**Cardiovascular risk management (CVRM) care**						
Healthy Heart program	15%**	€91		€0	**€89 (70-108)**	**0.000**
Primary care						
GP	0.5	€19	0.6	€22	€-4.2 (-13;4.3)	0.331
PN	2.5	€89	2.9	€105	**€-16 (-28;-4.0)**	**0.010**
TC	1.5	€28	1.3	€24	€3.7 (-0.5-7.9)	0.080
Hospital care						
Outpatient care	0.7	€71	0.8	€77	€-3.4 (-50;43)	0.886
Hospitalisation (days)	0.2	€123	0.1	€76	€50 (-101-201)	0.518
Dietician	1.5	€50	1.1	€35	€11 (-7.1;29)	0.230
Lifestyle coaching	0.7	€18	1.6	€33	€-16 (-65;33)	0.519
Medication usage CVRM		€230		€237	€11 (-30;52)	0.606
Laboratory costs CVRM		€81		€90	**€-9.0 (-16;-1.9)**	**0.013**
**Subtotal CVRM care**		*€801*		*€700*	*€106 (-71;293)*	*0.266*
**Other care**						
Primary care						
GP	4.7	€172	5.2	€190	€-22 (-52;7.1)	0.136
PN	2.0	€74	1.8	€67	€5.3 (-8.5;19)	0.448
TC	1.5	€27	1.2	€23	€2.8 (-7.4-13)	0.587
Home visits	1.2	€64	0.6	€32	**€35 (-0.07-71)**	**0.050**
Hospital care						
Outpatient care	3.0	€302	3.1	€311	€-2.8 (-69;63)	0.933
Hospitalisation (days)	0.7	€419	0.6	€297	€132 (-115;379)	0.294
Physiotherapist	13	€463	11	€415	€40 (-90;170)	0.544
Coaching	0.1	€7.5	0.1	€8.7	€2.6 (-11;5.4)	0.523
Home care						
Nursing aid	3.8	€210	2.3	€129	€101 (-182;385)	0.482
Domestic care	3.2	€81	3.6	€92	€-9.7 (-120;101)	0.862
Private care						
Private domestic	7.6	€168	7.0	€154	€9.9 (-162;182)	0.910
Informal care	7.9	€122	2.5	€38	€86 (-112;284)	0.393
**Subtotal other care**		*€2108*		*€1756*	*€379 (-163;922)*	*0.170*
**Total health care costs**		€2909		€2457	€484 (-110;1079)	0.110

Results are based on the pooled results of 100 multiple imputed datasets
Units are 'visits', unless otherwise specified
* adjusted for sex, age, baseline utility (EQ-5D-5L) and baseline costs
** percentage of participants
Bold numbers indicate mean adjusted difference between periods p≤0.05

CVRM: cardiovascular risk management; GP: general practitioner; PN: practice nurse TC: telephone consultations

Table S4. Costs of the Healthy Heart program per participant.

	Units	Unit price	Total costs
Personnel			
Intake	1	€58.00	€ 58.00
Group sessions	7	€14.50	€ 126.88
Individual in-depth consult	1	€58.00	€ 58.00
Return meeting	1	€18.13	€ 18.13
Individual evaluation meeting	1	€58.00	€ 58.00
TOTAL per participant			€ 319.00
TOTAL per participant including taxes			€ 385.99
Room rental rate/ hour*			
Intake	1	€7.52	€ 7.52
Group sessions	7	€1.88	€ 13.16
Individual in-depth consult	1	€7.52	€ 7.52
Return meeting	1	€1.88	€ 1.88
Individual evaluation meeting	1	€7.52	€7.52
Coffee and tea	11	€1.00	€11.00
TOTAL per participant			€ 48.60
TOTAL per participant including taxes			€ 434.59
Costs given 8 participants per group.<br>Rate lifestyle coach (per hour) € 58.00<br>*mean costs of 2 different locations			

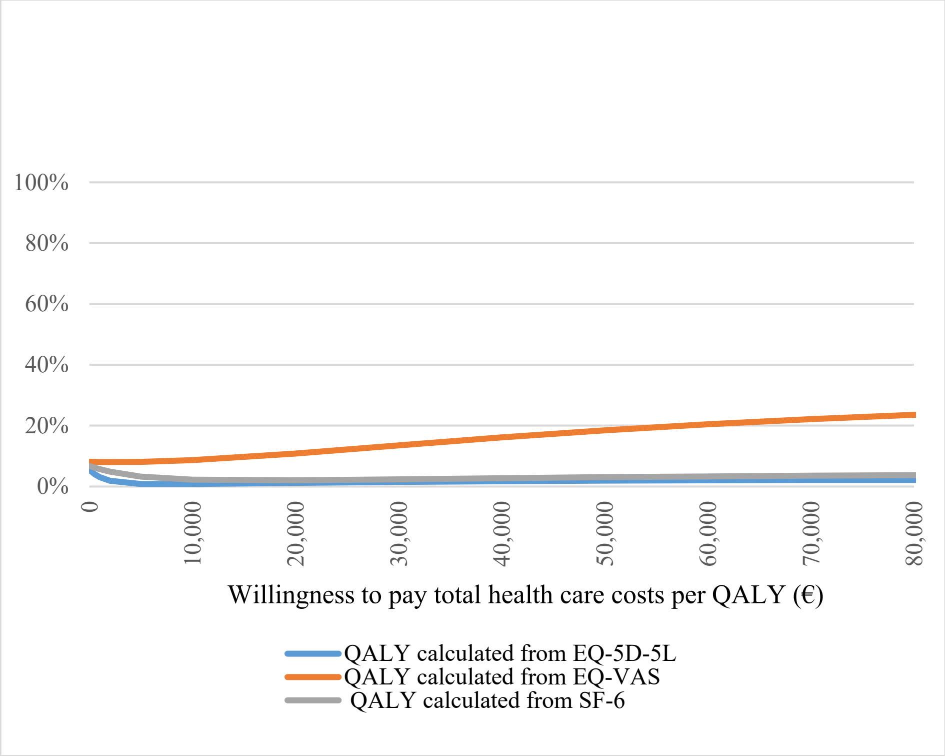

Figure S1. Cost-effectiveness acceptability curve.
The probability (%) that offering the Healthy Heart program is cost effective in a high cardiovascular risk population versus not offering the program, depending on willingness-to-pay for **total health care.**

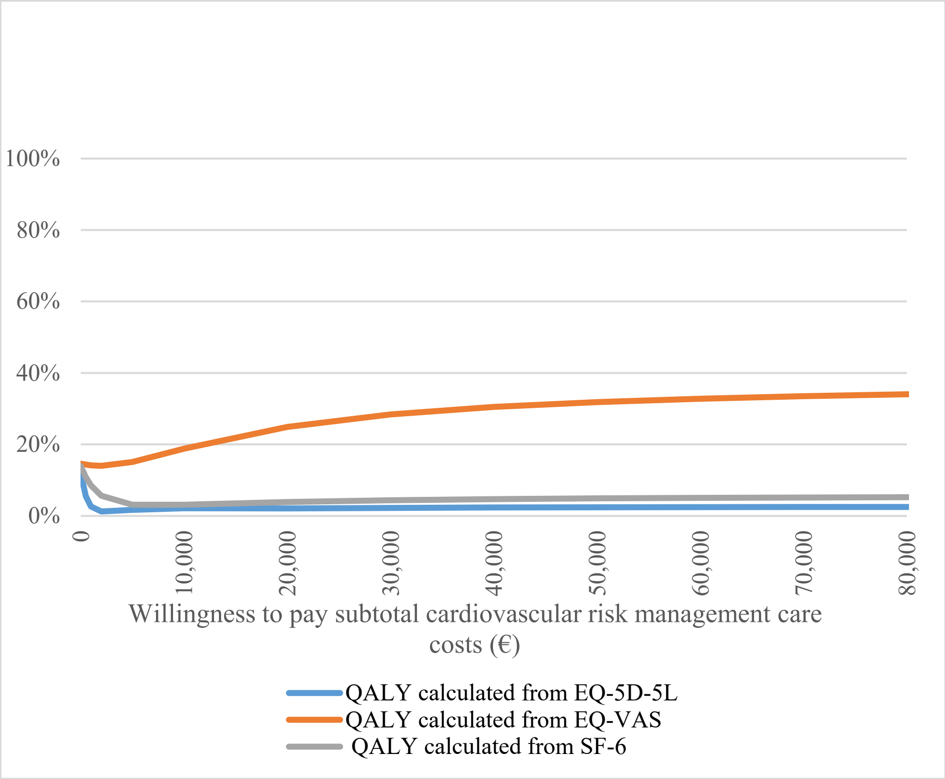

Figure S2. Cost-effectiveness acceptability curve.
The probability (%) that offering the Healthy Heart program is cost effective in a high cardiovascular risk population versus not offering the program, depending on willingness-to-pay for **cardiovascular ris.**
